# Enhanced In Vitro Expression of Filaggrin and Antimicrobial Peptides Following Application of Glycosaminoglycans and a Sphingomyelin-Rich Lipid Extract

**DOI:** 10.3390/vetsci9070323

**Published:** 2022-06-27

**Authors:** Sergi Segarra, Tanesha Naiken, Julien Garnier, Valérie Hamon, Nathalie Coussay, François-Xavier Bernard

**Affiliations:** 1 R&D Bioiberica S.A.U., 08950 Esplugues de Llobregat, Spain; 2 Bioalternatives, 86160 Gençay, France; t.naiken@bioalternatives.com (T.N.); j.garnier@bioalternatives.com (J.G.); valerie.hamon.de.almeida@qima.com (V.H.); nathalie.coussay@qima.com (N.C.); fxb@bioalternatives.com (F.-X.B.)

**Keywords:** filaggrin, antimicrobial peptides, canine atopic dermatitis, glycosaminoglycans, sphingolipids, sphingomyelin, β-defensin, hyaluronic acid

## Abstract

Filaggrin is an epidermal protein involved in skin barrier formation and hydration, whose expression is altered in canine atopic dermatitis (CAD). CAD patients also present an abnormal immune response with an altered expression of antimicrobial peptides (AMPs), such as β-defensins and cathelicidins. Sphingolipids and glycosaminoglycans (GAGs) have been reported to improve the skin barrier in several animal species, including dogs. Our objective was to evaluate the in vitro effects of a sphingomyelin-rich lipid extract (LE), a hyaluronic acid-rich GAG matrix, and their combination, on the expression of filaggrin and human β-defensin 2 (hBD-2). Filaggrin expression was quantified in a reconstructed human epidermis (RHE), and hBD-2 in normal human epidermal keratinocyte (NHEK) cultures. LE and GAGs were tested at 0.02 mg/mL, with or without adding a cytokine mix. A significant increase in mean hBD-2, compared to the control (99 pg/mL) was achieved with LE (138 pg/mL) and LE+GAGs (165 pg/mL). Filaggrin increased with GAGs (202% ± 83) and LE (193% ± 44) vs. the stimulated control, but this difference was statistically significant (*p* < 0.05) only with LE+GAGs (210% ± 39). In conclusion, the tested GAGs and LE enhance filaggrin and AMP expression in vitro, which might benefit CAD patients if applied in vivo.

## 1. Introduction

Canine atopic dermatitis (CAD) has been defined as a prevalent, genetically predisposed, chronically relapsing, progressive, pruritic, and inflammatory skin disease with characteristic clinical features and well-defined breed predispositions [1,2]. Recent advances indicate that it is actually a multifactorial and complex inflammatory syndrome [3]. Its pathogenesis is not yet completely understood, but it involves several factors leading to epidermal barrier dysfunction, immune dysregulation, and dysbiosis of the skin [3,4,5].

The skin barrier plays a key role in CAD, and epidermal barrier dysfunction occurs in both human and CAD, allowing penetration of irritant substances, microbes, and environmental allergens. This, in turn, stimulates the local immune system and induces a Th2- immune response [6,7,8]. One of the main targets of the multimodal approach to CAD management is restoring epidermal barrier function and integrity [4]. Although it is still not clear whether the defective skin barrier is pre-existing or is secondary to allergic inflammation, it has been suggested that some of the skin barrier anomalies may develop secondary to the underlying skin inflammation [9]. Lipid alterations have been reported in CAD with decreased levels of free fatty acid and ceramides in the *stratum corneum* (SC) [6].

Filaggrin is one of the most important epidermal proteins involved in the keratinization process. An abnormal catabolism of filaggrin occurs in atopic dermatitis and has been suggested as a cause for an abnormal skin barrier. Its expression can be modulated by genetics but also by inflammation [10,11,12,13]. Filaggrin distribution in the skin of dogs is similar to that of human and mouse skin [14]; however, compared to humans, the knowledge concerning the role of filaggrin in CAD is limited, and some conflicting results have been published when it comes to changes in its expression in atopic canine skin [15]. In dogs, atopic skin has been reported to feature lower filaggrin expression [16,17] as well as increased filaggrin-metabolizing enzyme activity [10]. In addition, disrupted profilaggrin degradation into filaggrin has a negative impact on barrier function and on the normal keratinization process. Moreover, reduced breakdown of filaggrin may contribute to decreased formation of natural moisturizing factors (NMFs). This, in turn, negatively affects skin hydration and UV light protection [10,18,19,20].

The affected atopic skin is frequently complicated by secondary microbial infections with *Staphylococcus pseudintermedius* and *Malassezia pachydermatis*. For this reason, the management of atopic dermatitis becomes even more challenging [7,21]. In fact, secondary infections can trigger relapses in patients with CAD that had been controlled [1]. Antimicrobial peptides (AMPs) are small immuno-modulatory proteins that have antimicrobial activity against bacteria, fungi, and viruses. AMPs also modulate innate and adaptive immune responses. In the skin of atopic dogs and people, there is an alteration in the expression of AMPs, including β-defensins and cathelicidins [20,22,23,24,25,26]. More specifically, in people, lower concentrations of the AMP human β-defensin 2 (hBD-2) have been reported in skin atopic patients, and a deficiency in AMP has been reported as a potential reason explaining the susceptibility to bacterial skin infection in these patients [27]. In dogs, a lower expression of β-defensin genes has been described in both lesional and non-lesional skin from CAD patients compared to normal dogs [28]. AMPs are, therefore, elements to take into consideration within the etiopathogenesis of atopic dermatitis and perhaps also when approaching treatment targets.

Sphingolipids are essential components of the eukaryotic cells’ plasma membrane, and they form the multilamellar water barrier in the SC of the epidermis, contributing to the epidermal permeability barrier function. Ceramides are the main epidermal sphingolipids, and decreased ceramide content in the epidermis leads to water loss and skin barrier dysfunction in dogs and in people [29,30,31]. On the other hand, hyaluronic acid (HA) is a glycosaminoglycan (GAG) and a major component of skin extracellular matrix. It is involved in the inflammatory response, angiogenesis, and tissue regeneration process, and it plays a key role in wound healing processes, including hemostasis, inflammation, cell proliferation, and remodeling [32,33]. 

Prior studies conducted using a lipid extract (LE) with a high content of sphingomyelin (Biosfeen^®3^, Bioiberica S.A.U., Palafolls, Spain), either alone or in combination with a HA-rich GAG matrix ingredients (Dermial^®^, Bioiberica S.A.U., Palafolls, Spain), describe their beneficial effects on skin health. More specifically, the application of this LE led to increased levels of ceramides and the number of lamellar bodies [34] in an in vitro model of skin equivalents, and when used in vivo, improvements in clinical signs were seen in a canine model of atopic dermatitis using a colony of high-IgE, experimentally sensitized atopic beagles [35]. Moreover, previous in vitro testing also supports the effects of this GAG matrix, showing enhanced proliferation and migration of fibroblasts and migration of keratinocytes, as well as increased elastin production and skin hydrating capacity [36,37,38].

The objective of these studies was to evaluate the in vitro effects of the abovementioned GAGs and LE, and their combination, on the expression of filaggrin and AMPs in order to better characterize their mechanisms of action and to further explore the potential beneficial effects of such products on skin health, especially in companion animals.

## 2. Materials and Methods

### 2.1. Test Compounds

Two products were tested in these studies: a sphingomyelin-rich lipid extract (LE; Biosfeen^®3^, Bioiberica, S.A.U., Palafolls, Spain) and a source of GAGs (Dermial^®^, Bioiberica, S.A.U., Palafolls, Spain) containing a high concentration of HA (60–75%), dermatan sulfate, and collagen. Both products were used at non-cytotoxic concentrations, which were selected based on a prior cell viability MTT assay.

### 2.2. Culture of Primary Keratinocytes under Basal Conditions

Normal human epidermal keratinocytes (NHEK) were obtained from surgical samples of healthy chest skin as previously described [39]. The use of these samples for research studies was approved by the Ethical Committee of the Poitiers Hospital (Poitiers, France). The cells were seeded in 96-well plates and cultured at 37 °C and 5% CO_2_ for 24 h in keratinocyte serum free medium supplemented with 0.25 ng/mL epidermal growth factor, 25 µg/mL pituitary extract, and 25 µg/mL gentamycin (Invitrogen Life Technologies, Carlsbad, CA, USA) at 37 °C and 5% CO_2_. Then, they were treated or not (control) with the test compounds (LE and GAGs), alone or in combination, at 0.02 mg/mL each. In order to simulate stimulated conditions (skin inflammation), a cytokine mix containing oncostatin M (OSM; R&D systems, Minneapolis, MN, USA), interleukin 17 (IL-17; R&D systems, Minneapolis, MN, USA), and tumor necrosis factor α (TNF-α, R&D systems, Minneapolis, MN, USA), at 5 ng/mL each, was added to the medium (stimulated control). The cells were then incubated for 72 h at 37 °C and 5% CO_2_. All experimental conditions were performed in triplicate.

### 2.3. Culture of Reconstructed Human Epidermis under Cytokine Mix–Stimulated Conditions

Reconstructed human epidermis (RHE) samples were prepared as previously described [40]. Briefly, suspensions of primary human keratinocytes from surgical samples of pediatric foreskins were cultured on 0.5 cm^2^ polycarbonate culture inserts (Millipore, Molsheim, France) in Epilife medium (Invitrogen Life Technologies, Carlsbad, CA, USA) supplemented with Epilife supplements and then transferred to the air–medium interface for 10 days and grown in Epilife medium (Invitrogen Life Technologies, Carlsbad, CA, USA) supplemented with 1.5 mmol calcium chloride and 50 µg/mL ascorbic acid. The 10-day-old RHE samples were then placed in a culture medium containing or not (control) the test compounds (LE and GAGs; systemic application), alone or in combination, at 0.02 mg/mL each, and preincubated for 24 h at 37 °C and 5% CO_2_. Then, the RHE samples were stimulated with a cytokine mix of interleukin 4 (IL-4; R&D systems, Minneapolis, MN, USA), IL-13 (R&D systems, Minneapolis, MN, USA), IL-22 (R&D systems, Minneapolis, MN, USA) and TNF- α (R&D systems, Minneapolis, MN, USA) at 3 ng/mL each, and the treatment with the test compounds was renewed or not (stimulated control). The RHE samples were further incubated for 48 h at 37 °C and 5% CO_2_. A non-stimulated and non-treated control condition was performed in parallel. All experimental conditions were performed in triplicate.

### 2.4. ELISA Test

After incubation, the culture supernatants were collected for quantification of hBD-2 release using a specific ELISA kit (BD-2 Human Development, PeproTech, Neuilly-sur-Seine, France) following the manufacturer’s instructions. The values were reported in pg/mL.

### 2.5. Immunofluorescence Labeling

The RHE samples were washed and fixed with formaldehyde solution. Fixed tissues were dehydrated with increasing ethanol concentrations and embedded in paraffin, and sections were carried out using a microtome (5 µm thickness). The sections were deparaffinized and incubated at 92 °C and pH 6 in a retrieval target solution in order to optimize antigen–antibody interaction. After saturation using a phosphate buffered saline solution (PBS)-Tween-5% milk solution, the sections were incubated at room temperature for 1 h with anti-filaggrin (Santa Cruz, Dallas, TX, USA) antibody. The binding sites recognized by the primary antibody were then revealed with a secondary fluorescent antibody (goat anti-mouse Alexa 488; Molecular probes, Eugene, OR, USA). Nuclei were labeled with propidium iodide (Sigma-Aldrich, Saint-Louis, MO, USA). Sections were observed using a NIKON E400 microscope. The images were captured using a NIKON DS-Ri1 and processed with NIS-Elements 4.13.04 software (Nikon, Tokyo, Japan). The fluorescence intensity and the surface area of the epidermis were measured using ImageJ software. The values of fluorescence intensity were normalized to the total epidermis surface area and reported as arbitrary units (AUs).

### 2.6. Statistical Methods

All results are expressed as mean ± SEM. The inter-group comparisons were performed by an unpaired Student’s *t*-test. Results were considered as significant when *p* < 0.05. The *p* values were as follows: * *p* < 0.05.

## 3. Results

### 3.1. Antimicrobial Peptide Expression

**Figure 1:**
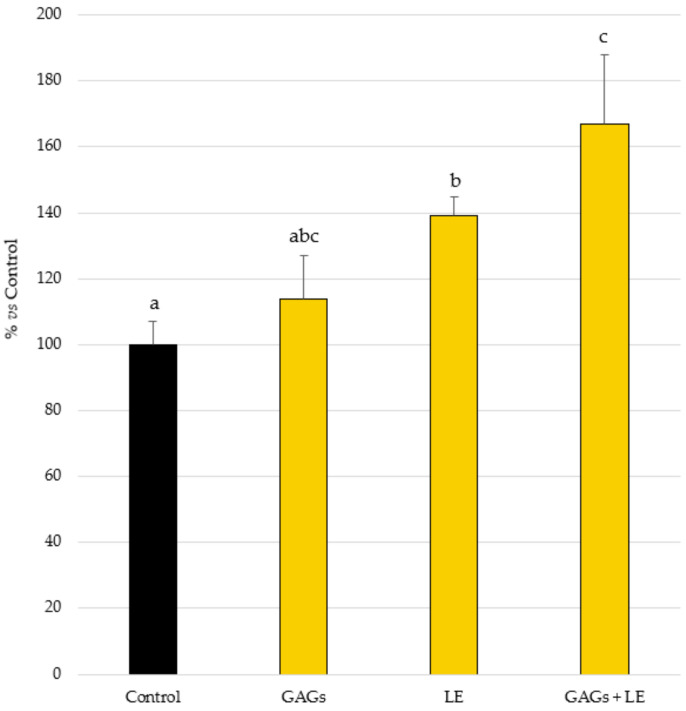
β-defensin 2 (hBD-2) production by normal human epidermal keratinocytes treated with cytokine mix (OSM + IL-17 + TNF-α at 5 ng/mL each) adding the test compounds (LE and GAGs), alone or in combination, at 0.02 mg/mL each, or not (Control). Mean ± SEM values of % of basal control are shown. Different letters indicate statistically significant differences.

A beneficial impact of the tested products was observed for AMP expression. More specifically, under basal conditions, a significant increase (*p* < 0.05) in mean hBD-2 production compared to the Basal Control (99 pg/mL; 100%) was achieved with LE (138 pg/mL; 139%) and the combination of LE with GAGs (165 pg/mL; 167%) (Figure 1). On the other hand, although it reached higher levels of hBD-2 release, the application of GAGs alone did not achieve a significant effect compared to the Control. As expected, the cytokine mix induced a marked hBD-2 release (>10,000 pg/mL; *p* < 0.001 vs. Basal Control).

### 3.2. Filaggrin Expression

**Figure 2:**
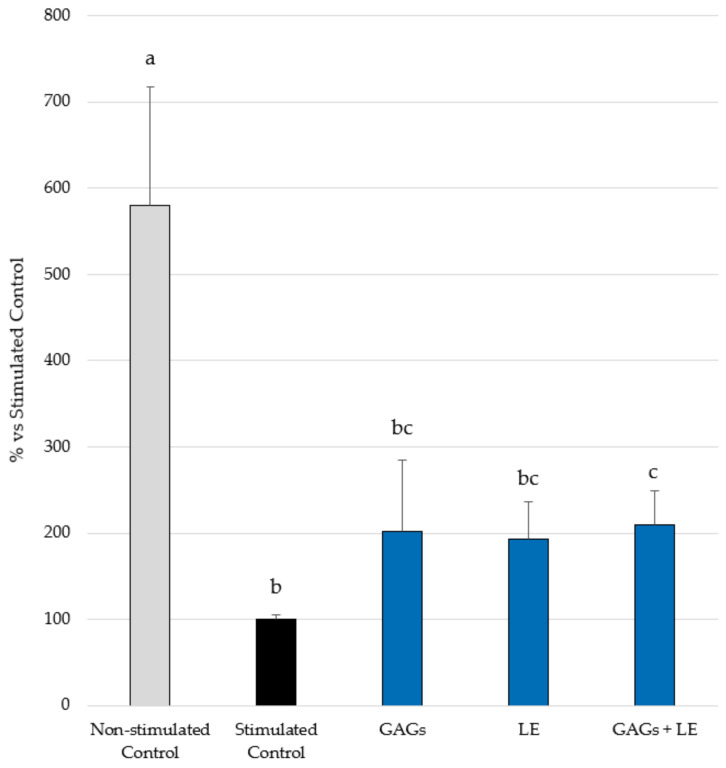
Filaggrin expression in reconstructed human epidermis (RHE) under cytokine mix (IL-4 + IL-13 + IL-22 + TNF-α at 3 ng/mL each)-stimulated conditions and treated or not (Stimulated Control) with the test compounds (LE and GAGs), alone or in combination, at 0.02 mg/mL each. Mean ± SEM values of % of stimulated control are shown. Different letters indicate statistically significant differences.

As expected, the Stimulated Control induced a strong inhibition of filaggrin expression (100%) compared to the Non-stimulated Control (mean ± SEM = 580% ± 137; *p* < 0.05). When the effect of LE and GAGs was evaluated, both products led to increased filaggrin expression. However, although higher levels were obtained with GAGs (202% ± 83) and LE (193% ± 44) compared to the Stimulated Control, this difference was statistically significant (*p* < 0.05) only with the combination of LE and GAGs (210% ± 39) (Figure 2). In the case of GAGs, this might be explained by the greater SEM.

**Figure 3:**
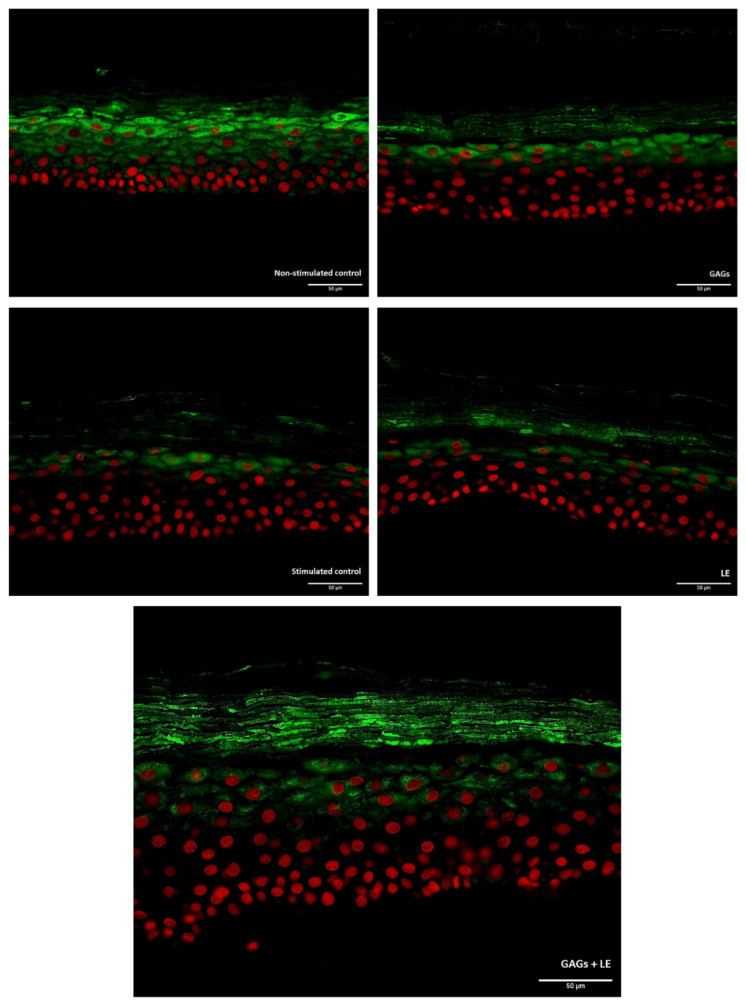
Immunofluorescent staining of filaggrin (green fluorescence) in reconstructed human epidermis (RHE) under cytokine mix (IL-4 + IL-13 + IL-22 + TNF-α at 3 ng/mL each)-stimulated conditions and treated or not (Stimulated Control) with the test compounds (LE and GAGs), alone or in combination, at 0.02 mg/mL each. Scale bar: 50 µm. Original magnification ×40.

When sections of the RHE samples were observed under the microscope, the impact on filaggrin expression of the tested products could also be seen (Figure 3).

## 4. Discussion

In the past, CAD was considered a histamine-driven type I hypersensitivity triggered by inhalant allergens, with IgE being a key player in the pathogenesis. Nowadays, however, it is seen as a very complex multifactorial syndrome, and it is well established that the skin barrier plays a key role [5,41]. That is why targeting the skin barrier and aiming at restoring it seems like an adequate treatment approach. This study reports there is a beneficial effect of an LE, with or without GAGs, on AMP expression, and an enhanced filaggrin expression with the combination of LE and GAGs. Therefore, this could help target critical elements in the etiopathogenesis of CAD. Given the positive impact of these compounds on such key factors involved in skin health, and particularly CAD, patients suffering from such conditions might benefit from their application as part of the multimodal treatment approach.

Lipid metabolism is key to preserving the integrity and function of the epidermal barrier [42]. The application of lipid-containing topical products, such as shampoos, sprays, and spot-ons, has been recommended as part of the multimodal therapeutic approach for the chronic management of CAD, with the aim of restoring epidermal barrier function and integrity [1]. This study reports that the tested compounds have an enhancing effect on filaggrin expression. Filaggrin and its metabolites are directly involved in maintaining skin barrier function and hydration. Since alterations in filaggrin metabolism can lead to an abnormal skin barrier [10], the use of such products could contribute to improving the management of CAD patients. A possible explanation for this positive impact on filaggrin expression might be the ability of this LE to act on the inflammatory process occurring in the skin [35], provided that filaggrin expression can also be modulated by inflammation [11]. Previous publications describe how other interventions, such as probiotics, have been tested with the aim of providing beneficial effects for CAD by enhancing filaggrin expression, without being successful [16]. A beneficial effect on skin inflammation could also be driven by the HA contained in the tested GAG matrix, based on prior observations [33,36].

In addition, atopic dermatitis patients have a higher risk of developing skin infections [21]. In dogs, there is an increased risk of recurrent microbial skin infections. The application of some topical antimicrobial products has been reported to lead to irritation or drying of the skin and thus exacerbate epidermal barrier dysfunction and adversely affect disease management [1]. The reduced AMP expression in atopic human patients has been suggested as a cause for recurrent skin infections; as also happens in dogs, these patients have an increased risk of developing secondary bacterial pyoderma and *Malassezia* dermatitis [22,27]. A reduced expression of AMPs may also be involved in the pathogenesis of CAD [13], and the reduced levels of AMPs and altered filaggrin metabolism occurring in the impaired atopic skin barrier provide a favorable environment for bacterial colonization [21]. Therefore, minimizing the development of secondary infections is also a target of the CAD proactive approach [4], hence the importance of the positive effect observed in this study in NHEK with the GAGs and LE tested. 

It is also worth mentioning that, in this study, hBD-2 production was quantified in NHEK, and one of the recent advances in our knowledge about atopic dermatitis over the past few years is that keratinocytes are no longer considered just a physical inert barrier. Instead, they are described as key players in the interaction between the nervous system and the immune cells [4].

In veterinary dermatology, the topical application of lipid-based formulations aimed at improving skin barrier dysfunction has been previously investigated in several studies in dogs [43,44,45,46,47,48,49,50,51,52]. The combination of the above-mentioned GAGs and LE used in the studies reported here has already been tested in CAD patients, leading to an attenuation of the clinical worsening induced by house dust mites [35]. The new data provided in this article describing an enhancement effect on the expression of filaggrin and AMPs could help explain the mechanisms of action behind the observed clinical benefits. A more thorough characterization of their mode of action should allow a more precise and rational application as well as open the door to further investigations.

In terms of potential clinical applications, CAD is a chronic disease that cannot be cured. In CAD patients, lifelong management is necessary, and treatment usually follows a multimodal approach. One of the main targets is to address all potential contributing flare factors of disease, and whether this management is successful or not will depend on applying a tailored management strategy. This strategy needs to be affordable and doable by the pet owner, hence the importance of adherence to treatment. Topical therapy thus becomes key to restoring epidermal barrier integrity and function [1]. This LE might, therefore, act as a bioactive moisturizer by enhancing skin barrier repair [53], and its use could fit within the proactive approach to CAD. Eventually, alone or in combination with other therapies, the use of this LE could contribute to preventing flares and reduce the need for rescue medication [4].

This study has some limitations that should be pointed out. First, our data show a positive impact of the sphingomyelin-rich LE, alone or in combination with the HA-rich GAG matrix, on hBD-2 expression, but an altered expression of the AMP cathelicidin has also been reported in atopic dogs [22]. This was not evaluated in this study and would be an interesting parameter to measure in future studies with these products. On the other hand, although the data presented herein are promising, it would be interesting to test compounds in vitro using canine cells instead of RHE. Lastly, further studies in patients with atopic dermatitis are warranted in order to being able to validate in vivo these in vitro observations. 

Administration of these LE and GAGs to companion animals, either applied orally or topically, might benefit dogs with atopic dermatitis or cats suffering from feline atopic syndrome. Depending on the outcome of such investigations, the potential of these products should be better defined.

## 5. Conclusions

In conclusion, the beneficial effects of the sphingomyelin-rich LE used in these studies, alone or in combination with an HA-rich GAG matrix, on filaggrin and AMP expression point towards the potential usefulness of these natural extracts in patients with atopic dermatitis in vivo and a possible preventive effect on disease onset.

## Data Availability

The datasets used and/or analyzed during the current study are available from the corresponding author on reasonable request.
